# Quality of Life of Healthcare College Students in Riyadh, Saudi Arabia

**DOI:** 10.7759/cureus.69811

**Published:** 2024-09-20

**Authors:** Arwa Talakey, Abrar Tounsi, Reyouf Alhusan, Maha Alabdely, Reem Alhamedi

**Affiliations:** 1 Periodontics and Community Dentistry, College of Dentistry, King Saud University, Riyadh, SAU; 2 College of Dentistry, King Saud University, Riyadh, SAU

**Keywords:** assessment, education, healthcare students, quality of life, well-being

## Abstract

Aim: The aim of this study was to assess the quality of life (QOL) and its influencing factors among healthcare college students in Riyadh, Saudi Arabia.

Methods: A cross-sectional study was conducted among healthcare students at King Saud University. A modified version of the Arabic World Health Organization Quality of Life-BREF (WHOQOL-BREF) questionnaire, including 25 questions, was used to assess students’ QOL. Data were collected through an online survey and analyzed using IBM SPSS Statistics version 26 (IBM Corp., Armonk, NY). Descriptive and regression statistics were used.

Results: A total of 547 healthcare students completed the questionnaire during the data collection period. Of all the students, 39.7% were from applied medical college, 62.5% were females, 98.9% were single, and 91.8% were non-smokers. Regarding academic status, 58.3% did not participate in any extracurricular activity, 42.6% had an academic advisor, and 80.4% had a grade point average (GPA) higher than 4.1. The average QOL score across all domains was moderately good (60.30 ± 16.0), whereas the physical domain showed the poorest score (46.89 ± 14.8) while the environmental domain had the greatest score (68.04 ± 20.6). The environmental domain was the only domain that showed significant differences between healthcare colleges (p < 0.001). Moreover, students who were male (p = 0.009), non-smokers (p = 0.023), reported living with family/friends (p = 0.031), and from families with high monthly income (p < 0.001) had significantly higher environmental QOL scores than their counterparts.

Conclusions: The study found that healthcare students had moderately good mean QOL scores. The environmental domain was the only QOL domain that showed significant differences between healthcare colleges with the greatest score among medical college students. Gender, smoking status, residence status, and family monthly income were found to have a strong association with students’ environmental QOL scores. Institutions need to focus on the regular measurement of students' QOL, which facilitates implementing strategies that can promote their overall QOL and subsequently positively impact their performance academically and practically.

## Introduction

The World Health Organization (WHO) defines quality of life (QOL) as “the individuals’ perception of their position in life in the context of the culture and value systems in which they live, and in relation to their goals, expectations, standards, and concerns” [1]. Based on this definition, physical and mental health, as well as social interactions and environmental circumstances, are all components of an individual’s QOL [1]. Maintaining the QOL of healthcare professionals is essential to ensure the delivery of high-quality healthcare, patient safety, and satisfaction. Therefore, it is important to consider the concept of QOL in healthcare practices and institutions.

Great attention has been drawn to psychological well-being among healthcare students, especially medical and dental students. Several studies have revealed significant levels of distress, nervousness, and depression among healthcare students [2-6]. Multiple factors may contribute to these conditions, including a massive curriculum, number of required assignments, deadlines, exams, workload, and dealing with patients [3]. Thus, these conditions may lead to poor QOL among healthcare students, which may compromise their academic performance, delivery of high-quality healthcare, and patient satisfaction.

The World Health Organization Quality of Life-BREF (WHOQOL-BREF) instrument is a commonly used, cross-cultural, multilingual questionnaire [7]. It may be used to evaluate differences in life quality between cultures and different groups within the same culture, and to track changes through time in reaction to life conditions changes [7]. The WHOQOL-BREF has been used in several studies carried out in Saudi Arabia to assess the QOL of healthcare students from medical and dental colleges [8-11]. The results of these studies revealed that gender could influence students’ QOL [8-11]. One study [11] was conducted among medical students solely and found that some sociodemographic factors were significantly associated with QOL.

Previous studies [8-11] on Saudi students focused on assessing QOL among medical or dental students individually, without comparison or assessment of differences among other healthcare specialties. Moreover, the role of different factors that may impact students’ self-evaluation of their QOL has not been comprehensively assessed. Thus, it would be of interest to assess QOL among students from different healthcare specialties and investigate the different factors that can affect it, to enhance their QOL that would positively affect their academic performance and future career practice.

Understanding the QOL of healthcare students and different factors that can influence it is essential for university administrators to best serve students and introduce interventions that could improve their QOL and subsequently their performance. Therefore, this study aimed to assess the QOL of healthcare students using the WHOQOL-BREF instrument and determine the role of sociodemographic factors (gender, marital status, residence, and family monthly income), health habits (smoking status and physical activity), extracurricular activity, and academic status on their QOL.

This article was previously presented as a poster at the 35th Saudi International Dental Conference (SIDC 2024) held on 18th-20th January 2024.

## Materials and methods

Study design and participants

The study adopted a cross-sectional design. The target population was healthcare college students at King Saud University (KSU), Riyadh, Saudi Arabia. Based on the WHO guidance for the smallest sample size required for a prevalence investigation, the estimated sample size of this study was 384 people using a margin of error of 5%, a confidence interval of 95%, and a standard deviation of 0.5 [12].

The questionnaire was distributed between September 25, 2022, and October 8, 2022, and two reminders were sent within this data collection timeframe. This period was specifically selected to avoid the influence of exam stress on students’ responses, as it was a month after the start of the new academic year and two weeks before their exams started.

Inclusion and exclusion criteria

Students included in the study were those currently registered students in one of the healthcare colleges (Medicine, Dentistry, Pharmacy, Applied Medical Sciences, and Nursing) at KSU. Interns, part-time students, those on medical leave, students who deferred their studies to a later semester, and those from non-healthcare colleges were excluded from the study. Students were recruited using a convenience sampling technique.

Ethical approval

The Institutional Review Board of Health Sciences Colleges Research on Human Subjects at King Saud University ethics committee gave its approval to the study protocol (E-22-7078). Subjects voluntarily consented to participate in the study.

Data collection

Data were collected using an electronic self-administered questionnaire via WhatsApp (Meta, Menlo Park, CA). Before being enrolled in the study, all subjects provided their informed consent. Students who accepted to take part in the study were asked to respond to the questionnaire based on their feelings during the previous two weeks. As the study was assessing the QOL of healthcare students focusing on their current experiences, to avoid the influence of exam stress on their responses, the WHO two-week timeframe recommendation was adopted for its appropriateness.

Questionnaire

Healthcare college students completed a self-administered questionnaire to evaluate their QOL, which included questions regarding sociodemographic characteristics (gender, marital status, residence, and family's monthly income), health habits (smoking status and physical activity), academic status (college name, grade point average (GPA), extracurricular activity, and having an academic advisor), and questions from the Arabic version of the WHOQOL-BREF. The questionnaire has been translated into many languages and used in developed and developing countries. Several studies have assessed the validity and reliability of the Arabic version of the tool in Arabic-speaking populations [13].

The Arabic WHOQOL-BREF [7] was used with some modification (only 25 questions were included), and permission for using it was obtained from the WHO (ID: 389593). The questionnaire comprised two independent questions (overall QOL and satisfaction with health) and questions related to four domains: physical, psychological, social relationships, and environmental [7].

Each domain consists of different facets, where a facet can be a description of a behavior (e.g. walking), a state of being (e.g. fatigue), a capacity or potential (e.g. ability to move), or a subjective perception or experience (e.g. pain). Moreover, different domains consist of different numbers of facets and questions, where the physical domain comprises three main facets “pain and discomfort, energy and fatigue, and sleep and rest” and seven questions. The psychological domain includes five facets and six questions related to feelings, thinking, concentration, self-esteem, and appearance. The social relationships domain in our study was modified to include two facets composed of two questions only “personal relationships and social support” while the sexual activity facet was excluded due to cultural acceptability reasons. Finally, the environmental domain comprises eight facets and questions related to security, financial resources, health and social services availability, and other characteristics. The definition and detailed description of each domain facet can be found in the WHOQOL User Manual [7].

Scoring system

Each item in the tool is scored using a five-point Likert scale, with 1 being the lowest rating (very poor/very dissatisfied/none/never) and 5 being the highest rating (very good/very satisfied/extremely/always). The scale for domain scores is positive (i.e. higher ratings indicate a greater QOL), except for three negatively framed questions (“To what extent do you feel that [physical] pain prevents you from doing what you need to do?,” “How much do you need any medical treatment to function in your daily life?,” and “How often do you have negative feelings such as blue mood, despair, anxiety, depression?”), where the recoding of scores was conducted to transform them into positively framed questions. The domain score was determined using the average score of the questions included in each domain. Next, to create domain scores equivalent to the scores used in the WHOQOL-100, average scores were multiplied by 4, and after that, the score was converted to a scale of 0-100 using the following equation [7]: Transformed score = (score - 4) x (100 ÷ 16).

As WHOQOL-BREF is the short version of the WHOQOL-100, this transformation of converting the lowest and highest possible scores to zero and 100, respectively, was necessary and recommended to make domain scores comparable with the scores used in the original WHOQOL-100.

Statistical analysis

The data analysis was performed using IBM SPSS Statistics version 26 (IBM Corp., Armonk, NY). Descriptive statistics of categorical variables are presented as frequencies and percentages, while means and standard deviations (SD) are used to present numerical variables. The Shapiro-Wilk test was performed to evaluate the normality of the QOL domain scores. As the normality assumption of QOL domain scores was not met, the Mann-Whitney U test was used to compare mean scores of QOL domains between dichotomous variables (gender, marital status, residence, smoking status, physical activity, extracurricular activity, and academic advisor). Kruskal-Wallis test was implemented to compare the mean score of QOL domains for variables with three or more category groups (healthcare colleges, family monthly income, and GPA); when a significant difference was found, a post hoc test (Dunn test) was utilized to assess intergroup mean differences. The threshold for statistical significance was determined as p-value <0.05. The association between different factors (sociodemographic factors, health habits, and academic status) and environmental domain was assessed using multiple linear regression.

## Results

A total of 547 healthcare students completed the questionnaire during the data collection period. Table 1 shows the sociodemographic factors, health habits, and academic status distribution among the participants stratified by healthcare colleges. Of all the students, 39.7% were from applied medical college, with significant differences across healthcare colleges in the distribution of gender, marital status, smoking status, and academic status (p < 0.05). Most of the participants were female (342 students, 62.5%), single (541 students, 98.9%), and non-smokers (502 students, 91.8%). Regarding academic status, 319 (58.3%) did not participate in any extracurricular activity, 425 (42.6%) had an academic advisor, and 440 (80.4%) had a GPA higher than 4.1 (Table 1).

**Table 1:** Distribution of study variables stratified by healthcare colleges (N = 547). GPA: grade point average.

Study variables		Total (N = 547), N (%)	Medical College (N=101), N (%)	Dental College (N = 69), N (%)	Pharmacy College (N = 76), N (%)	Applied Medical College (N = 217), N (%)	Nursing College (N = 84), N (%)	P-value
Sociodemographic factors								
Gender	Male	205 (37.5)	49 (24.0)	20 (9.0)	26 (13.0)	100 (49.0)	10 (5.0)	<0.001
	Female	342 (62.5)	52 (15.0)	49 (14.0)	50 (15.0)	117 (34.0)	74 (22.0)	
Marital status	Single	541 (98.9)	100 (18.0)	69 (13.0)	75 (14.0)	217 (40.0)	80 (15.0)	0.009
	Married	6 (1.1)	1 (17.0)	0 (0.0)	1 (17.0)	0 (0.0)	4 (66.0)	
Residence	Alone	33 (6)	4 (12.1)	4 (12.1)	7 (21.2)	11 (33.3)	7 (21.3)	0.514
	With family or friends	514 (94)	97 (19.0)	65 (13.0)	69 (13.0)	206 (40.0)	77 (15.0)	
Family monthly income	<5000	80 (14.6)	7 (9.0)	7 (9.0)	15 (19.0)	34 (42.0)	17 (21.0)	0.130
	5000-10000	117 (21.4)	19 (16.0)	13 (11.0)	17 (15.0)	50 (43.0)	18 (15.0)	
	>10000	350 (64)	75 (21.0)	49 (14.0)	44 (13.0)	133 (38.0)	49 (14.0)	
Health habits								
Smoking status	Non-smoker	502 (91.8)	98 (20.0)	66 (13.0)	73 (14.0)	190 (38.0)	75 (15.0)	0.013
	Smoker	45 (8.2)	3 (7.0)	3 (7.0)	3 (7.0)	27 (60.0)	9 (19.0)	
Physical activity	No	306 (55.9)	57 (19.0)	45 (14.0)	42 (14.0)	120 (39.0)	42 (14.0)	0.452
	Yes	241 (44.1)	44 (18.3)	24 (10.0)	34 (14.1)	97 (40.2)	42 (17.4)	
Academic status								
Extracurricular activity	No	319 (58.3)	62 (19.0)	33 (10.0)	37 (12.0)	121 (38.0)	66 (21.0)	<0.001
	Yes	228 (41.7)	39 (17.0)	36 (16.0)	39 (17.0)	96 (42.0)	18 (8.0)	
Academic advisor	No	122 (57.4)	58 (47.0)	34 (28.0)	1 (1.0)	23 (19.0)	6 (5.0)	<0.001
	Yes	425 (42.6)	43 (10.0)	35 (8.0)	75 (18.0)	194 (46.0)	78 (18.0)	
GPA	2-3	15 (2.7)	0 (0.0)	0 (0.0)	0 (0.0)	12 (80.0)	3 (20.0)	<0.001
	3.1-4	92 (16.9)	15 (16.3)	0 (0.0)	4 (4.4)	53 (57.6)	20 (21.7)	
	>4.1	440 (80.4)	86 (19.5)	69 (15.7)	72 (16.4)	152 (34.5)	61 (13.9)	

On a scale of 0-100, the participants’ mean score was (60.30 ± 16.0). Among the four domains, the physical domain had the poorest mean QOL score (46.89 ± 14.8), while the environmental domain had the greatest mean QOL score (68.04 ± 20.6).

Table 2 presents the association between sociodemographic factors, health habits, academic status, and QOL among healthcare students. The mean physical score was significantly higher among healthcare students who reported practicing physical activity (48.8 ± 15.3,p = 0.010), participating in an extracurricular activity (48.4 ± 14.1, p = 0.035), and had an academic advisor (47.6 ± 14.9, p = 0.037). In the psychological domain, healthcare students with high family monthly income (>10000 Saudi riyal (SR)) had significantly higher mean scores than students with low family monthly income (<5000 SR) (66.0 ± 17.2 vs. 58.8 ± 19.6, p = 0.010). Moreover, students who reported practicing physical activity and participated in extracurricular activities had significantly higher mean scores than their counterparts (66.4 ± 18.9 vs. 62.9 ± 17.2, p = 0.010 and 66.7 ± 17.8 vs. 62.8 ± 18.1, p = 0.014, respectively). The social relationship domain mean score was significantly higher among healthcare students with high family monthly income (>10000 SR) than among those with low family monthly income (<5000 SR) (64.3 ± 26.2 vs. 55.6 ± 25.5,p = 0.010). Furthermore, healthcare students who reported living with family or friends and those participating in extracurricular activity had significantly higher mean scores than their counterparts (62.4 ± 26.5 vs. 52.7 ± 26.1, p = 0.049 and 66.8 ± 24.4 vs. 58.2 ± 27.4, p < 0.001, respectively). Finally, for the environmental domain, the mean score was significantly higher among healthcare students who were male, reported living with family or friends, and had high family monthly income (71.2 ± 21.0, p = 0.001; 68.6 ± 20.6, p = 0.022; and 71.8 ± 19.1, p < 0.001, respectively) compared to females (66.1 ± 20.2), those living alone (59.9 ± 20.6), and those with low family monthly income (59.5 ± 21.6). Moreover, the environmental domain was the only QOL domain that showed significant differences between the healthcare colleges (p < 0.001). Students from medical college had a significantly highest mean score of 75.6 ± 18.3 among other healthcare college students, including dental (65.6 ± 20.8, p = 0.019), pharmacy (65.8 ± 21.6, p* *= 0.020), applied medical sciences (68.8 ± 19.9, p = 0.043), and nursing college (61.1 ± 21.7, p < 0.001).

**Table 2:** Association between sociodemographic factors, health habits, and academic status and different domains of QOL among healthcare students. QOL: quality of life; GPA: grade point average.

Study variables		Physical domain, mean (SD)	P-value		Psychological domain, mean (SD)	P-value		Social relationships domain, mean (SD)	P-value	Environmental domain, mean (SD)	P-value
Sociodemographic factors											
Gender	Male	47.6 (15.1)	0.185		64.9 (17.9)	0.603		61.4 (26.3)	0.838	71.2 (21.0)	0.001
	Female	46.4 (14.8)			64.1 (18.2)			62.0 (26.7)		66.1 (20.2)	
Marital status	Single	46.9 (14.8)	0.951		64.4 (18.0)	0.862		62.0 (26.4)	0.368	68.1 (20.6)	0.931
	Married	46.4 (21.5)			66.0 (21.0)			50.0 (36.2)		66.7 (24.9)	
Residence	Alone	49.8 (19.0)	0.516		61.9 (18.0)	0.459		52.7 (26.1)	0.049	59.9 (20.6)	0.022
	With family or friends	46.7 (14.6)			64.6 (18.1)			62.4 (26.5)		68.6 (20.6)	
Family monthly income	<5000	47.1 (15.1)	0.695		58.8 (19.6)	0.010		55.6 (25.5)	0.010	59.5 (21.6)	<0.001
	5000-10000	46.0 (15.6)			63.7 (18.8)			58.7 (27.4)		62.5 (21.7)	
	>10000	47.1 (14.7)			66.0 (17.2)			64.3 (26.2)		71.8 (19.1)	
Health habits											
Smoking	Non-smoker	47.2 (14.9)	0.125		64.8 (17.8)	0.086		61.7 (26.7)	0.805	68.5 (20.1)	0.203
	Smoker	43.7 (14.7)			59.5 (19.8)			63.05 (25.4)		62.4 (25.8)	
Physical activity	No	45.4 (14.4)	0.010		62.9 (17.2)	0.010		60.1 (27.6)	0.158	67.05 (19.9)	0.108
	Yes	48.8 (15.3)			66.4 (18.9)			63.9 (25.0)		69.2 (21.5)	
Academic status											
Healthcare college	Medical College	46.8 (15.2)		0.190	65.5 (17.5)		0.089	64.9 (28.5)	0.357	75.6 (18.3)	<0.001
	College of Dentistry	43.3 (13.9)			62.4 (18.3)			61.2 (27.9)		65.6 (20.8)	
	College of Pharmacy	45.7 (14.8)			64.9 (18.0)			61.2 (24.5)		65.8 (21.6)	
	College of Applied Medical Sciences	47.8 (14.4)			66.0 (18.3)			62.3 (26.0)		68.8 (19.9)	
	College of Nursing	48.6 (16.4)			60.5 (17.6)			58.0 (26.5)		61.1 (21.7)	
Extracurricular activity	No	45.8 (15.4)	0.035		62.8 (18.1)	0.014		58.2 (27.4)	0.000	66.4 (21.7)	0.056
	Yes	48.4 (14.1)			66.7 (17.8)			66.8 (24.4)		70.2 (19.1)	
Academic advisor	No	44.6 (14.7)	0.037		63.01 (18.3)	0.394		61.3 (27.1)	0.877	69.4 (20.4)	0.383
	Yes	47.6 (14.9)			64.8 (17.9)			61.9 (26.4)		67.6 (20.7)	
GPA	2-3	47.4 (15.1)	0.935		63.1 (19.5)	0.426		57.5 (27.9)	0.569	60.0 (25.2)	0.332
	3.1-4	46.6 (15.3)			62.1 (18.4)			59.4 (28.2)		66.5 (20.8)	
	>4.1	47.0 (14.8)			65.0 (18.0)			62.5 (26.2)		68.6 (20.4)	

The crude associations between different factors (sociodemographic factors, health habits, and academic status) and environmental domain are shown in Table 3. Based on healthcare colleges, students from dental college (ß: -8.98; 95% CI: -15.07, -2.88), pharmacy college (ß: -8.65; 95% CI: -15.10, -2.19), and nursing college (ß: -10.49; 95% CI: -16.89, -4.09) were significantly associated with lower environmental scores than medical college students (p < 0.05). Furthermore, students who were female (ß: -5.00; 95% CI: -8.75, -1.25) and smokers (ß: -7.31; 95% CI: -13.60, -1.01) were significantly associated with lower environmental scores compared to students who were male and non-smokers (p < 0.05). On the other hand, students who reported living with family or friends (ß: 3.83; 95% CI: 0.35, 7.31) and those with high family monthly income (ß: 11.25; 95% CI: 6.40, 16.09) had significantly higher environmental scores than those who reported living alone and with low family monthly income (p < 0.05).

**Table 3:** Multiple linear regression between sociodemographic factors, health habits, and academic status and environmental domain. GPA: grade point average; SR: Saudi riyal.

Study variables	Crude associations	
	ß (95% CI)	P-value
Sociodemographic factors		
Gender		
Male	1.00 (Reference)	
Female	-5.00 (-8.75, -1.25)	0.009
Marital status		
Single	1.00 (Reference)	
Married	3.20 (-12.91, 19.31)	0.697
Residence		
Alone	1.00 (Reference)	
With family or friends	3.83 (0.35, 7.31)	0.031
Family monthly income		
<5000 SR	1.00 (Reference)	
5000-10000 SR	3.16 (-2.51, 8.83)	0.275
>10000 SR	11.25 (6.40, 16.09)	<0.001
Health habits		
Smoking status		
Non-smoker	1.00 (Reference)	
Smoker	-7.31 (-13.60, -1.01)	0.023
Physical activity		
No	1.00 (Reference)	
Yes	1.96 (-1.41, 5.33)	0.253
Academic status		
Healthcare colleges		
Medical College	1.00 (Reference)	
Dental College	-8.98 (-15.07, -2.88)	0.004
Pharmacy College	-8.65 (-15.10, -2.19)	0.009
Applied Medical Science	-5.24 (-10.45, -0.03)	0.049
Nursing College	-10.49 (-16.89, -4.09)	0.001
Extracurricular activity		
No	1.00 (Reference)	
Yes	2.96 (-0.48, 6.40)	0.092
Academic advisor		
No	1.00 (Reference)	
Yes	2.32 (-2.38, 7.02)	0.332
GPA		
2-3	1.00 (Reference)	
3.1-4	6.86 (-3.98, 17.69)	0.214
>4.1	8.16 (-2.29, 18.61)	0.126

## Discussion

Our study evaluated the QOL and the factors that affect it among healthcare students at KSU, Saudi Arabia. The study found that the mean QOL score among the four domains was 60.3 ± 16.0, which may be considered a moderately good QOL. Moreover, the lowest score was for the physical domain while the greatest score was for the environmental domain.

Regarding factors associated with QOL, students who reported living with family or friends, with high family monthly income, practicing physical activity, and participating in extracurricular activities had better QOL than their counterparts. These results are supported by previous studies that assessed some of the factors that influence QOL among university students [11,14]. High family income affects self-esteem, improves efficacy, and reduces frustration [11]. Living with family or friends facilitates stress coping and improves psychological well-being [11]. Moreover, physical activity directly influences physical self-esteem, while participation in extracurricular activities enables strong social networking and benefits physical and mental health [14,15]. Therefore, the QOL of healthcare students may have been positively affected given the great benefit of these factors.

In terms of the physical domain poor score, the domain includes facets related to pain and discomfort, energy and fatigue, and sleep and rest. Healthcare students spend a long time in their colleges between didactic and practical sessions, which could have led to physical exhaustion and limited time to engage in daily activities. Moreover, the excessive workload and stress that healthcare students face daily may have affected their sleep satisfaction and, subsequently, lowered their energy level to perform daily tasks and other chosen activities. Therefore, all these factors may have negatively affected the facets scores that constitute the physical domain. Even though students in different health specialties may be exposed to slightly different levels of workload and educational systems, no significant differences were found across QOL domains based on healthcare colleges, except for the environmental domain, which had the highest score across all other domains. Moreover, the highest environmental domain score was found among medical students, where their high family income, availability, and easier access to health information and services may be the contributing factors for this high score, as they form the majority of the environmental domain facets [16].

Regarding factors affecting the environmental domain, students who were male, non-smokers, reported living with family or friends, and with high family monthly income had higher environmental scores than their counterparts. Among university students, females have been found to have a lower QOL than males, which may be explained by the fact that females tend to recognize many situations and requirements as stressful, thus negatively affecting their QOL [17]. Additionally, smoking has been found to negatively impact QOL as it adversely affects sleep quality and is associated with higher anxiety and depression levels [18,19]. Finally, family atmosphere and high family income have been found to contribute to high QOL, as families with high incomes may have the time and financial resources to provide better investments, safer homes, and greater support to their offspring than those with low incomes [20,21].

The study findings provide recommendations for policy development and future research. They support earlier studies that suggest regular monitoring of students' QOL to improve their educational environment [8-11]. Additionally, the findings focus on healthcare college students, as their programs are considered stressful, challenging, and involve dealing with patients. Thus, these colleges should implement strategies to improve students’ QOL, which will have a positive impact on their academic performance, delivery of high-quality healthcare, and patient satisfaction. These strategies may include establishing counseling centers that can assist students with physical and psychological health issues [22]. Establishing sociocultural clubs that organize extracurricular activities and encourage student participation may assist in building personal relationships and social support. Additionally, incorporating physical activity breaks into weekly schedules may enhance students’ focus and reduce stress. Finally, advantages can be achieved at an institutional level, where focusing on improving students’ experiences, including taking into consideration physical, psychological, social, and environmental conditions, is of great importance to institutions in the modern context, especially with the considerable increase in the number of healthcare institutions in Saudi Arabia. Applying such strategies would help reduce the number of students who withdrew and build a sense of affinity with students. Thus, alumni will be positively inclined to their institutions, serve as effective ambassadors, and are more likely to encourage peers and family members to consider their institutions that will support future enrolment and financial revenues.

The study has some limitations that need to be taken into consideration. First, the use of a convenience sampling technique of healthcare students from one university may influence the generalizability of our findings. Therefore, further studies using random sampling techniques from multiple universities are recommended. In addition, students’ levels of study could not be compared in this study because not all healthcare colleges at KSU adhere to the same academic year leveling system, which may affect the QOL domains since senior and junior students might have different stress-coping abilities. Finally, females constituted a large proportion of our sample, which may shifted the results either way. However, regression analysis was conducted to overcome the confounding effect of gender and other variables associated with the outcome.

## Conclusions

This study found that healthcare students had moderately good mean QOL scores, with the poorest scores in the physical domain and the greatest scores in the environmental domain. The environmental domain was the only QOL domain that showed significant differences between healthcare colleges with the greatest score among medical college students. Gender, smoking status, residence status, and family monthly income were found to have a strong association with students’ environmental scores. Institutions need to focus on the regular measurement of students' QOL, which facilitates implementing strategies that can promote their overall QOL and subsequently positively impact their performance academically and practically.
